# Supplementary figures and images for: Apical spectrin organizes cortical actin filament bundles to pattern C. elegans cuticle ridges
Source: PLoS Genet. 2026 Jul 16;22(7):e1012236. doi: 10.1371/journal.pgen.1012236 (PMC13395344; doi:10.1371/journal.pgen.1012236)

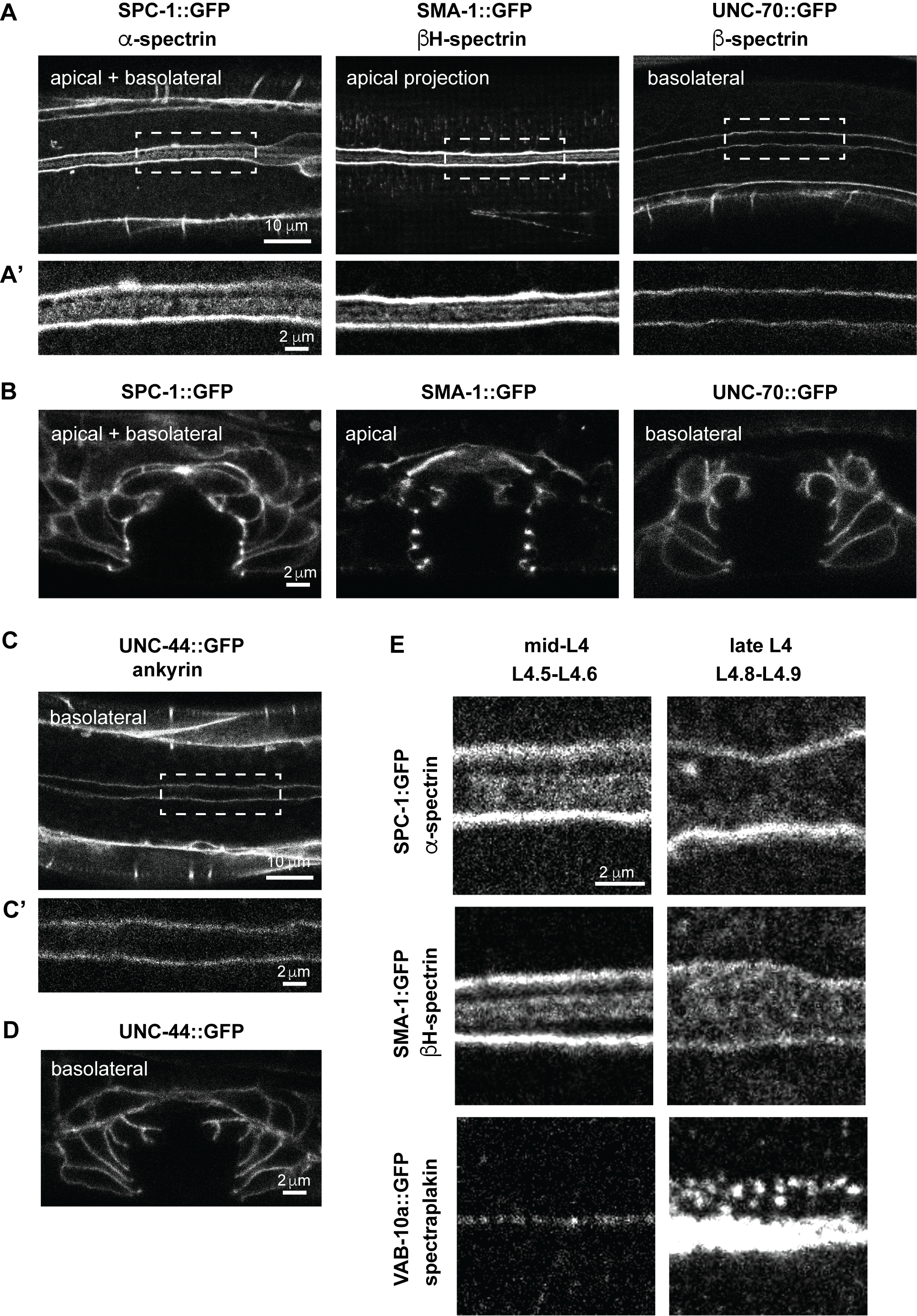

Supplement: S1 Fig — Confocal images of spectrin localization at: (A, A’) the apical hypodermis and seam and (B) midsection of a mid-L4 vulva tube. SMA-1::GFP localizes to apical membranes while UNC-70::GFP localizes to basolateral membranes; SPC-1::GFP localizes to all membranes. (C, C’, D) UNC-44::GFP (ankyrin) is basolateral similar to UNC-70. (E) Comparison of apical spectrin and spectraplakin patterns during alae-patterning at mid-L4 (left) vs after alae formation at late L4 (right). Spectrin begins to disperse by late L4, similar to actin [20], while VAB-10 signal increases due to CeHD formation. All images representative of at least n = 10 specimens imaged. See also Movies S1-S5. (TIF) [file pgen.1012236.s001.tif]

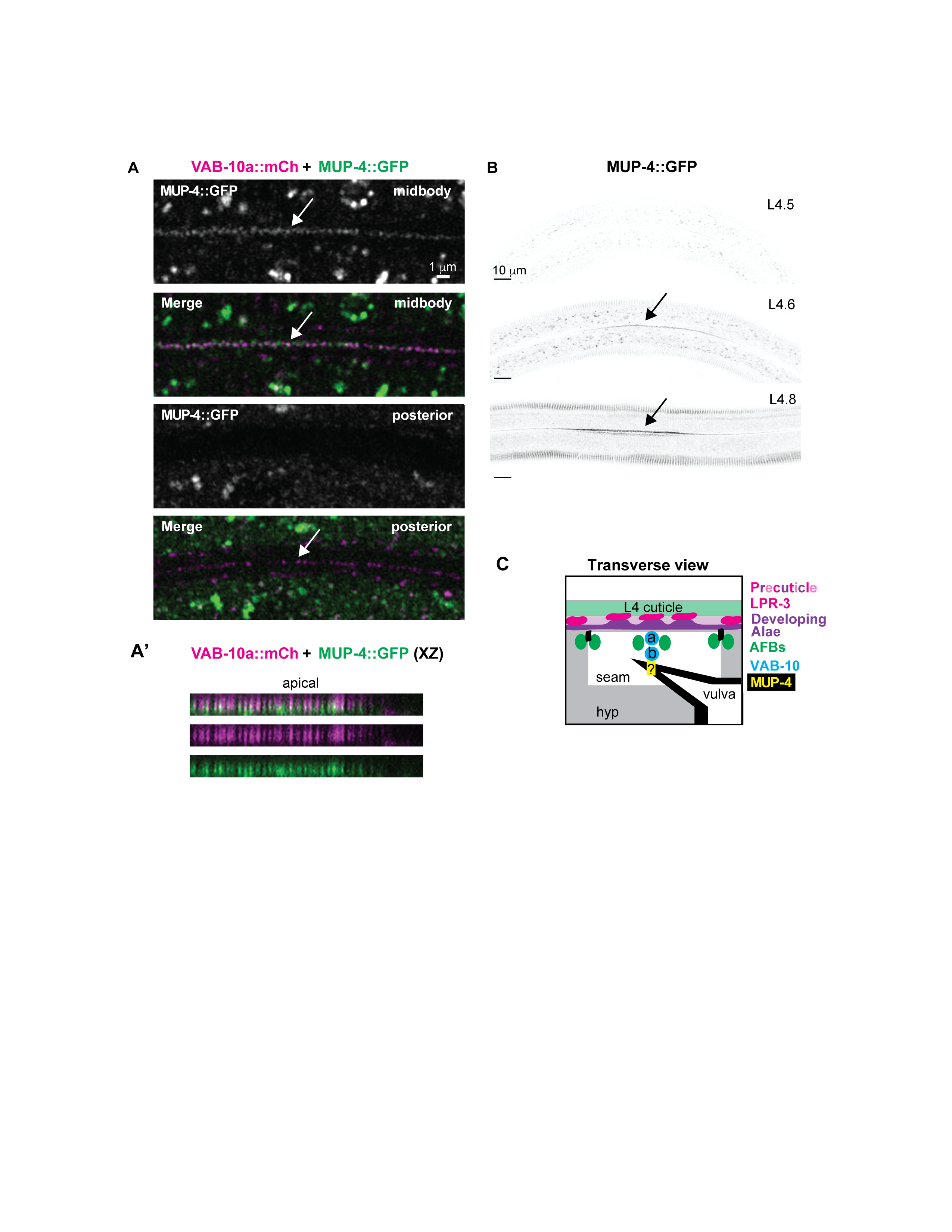

Supplement: S2 Fig — Single confocal slices showing the patterning of MUP-4::GFP at the seam at the indicated L4 substages. MUP-4 starts forming a linear pattern at the medial seam at approximately the L4.5 substage. By L4.8 the signal at the seam intensifies and extends from the middle towards the anterior and posterior ends of the worm but doesn’t localize to the two extreme ends of the worm. (D) Schematic summary. The precise architecture of CeHDs in the mid-body has not yet been investigated, but dorsal vulva cells are known to attach to both the seam and various sex muscles [56]. (TIF) [file pgen.1012236.s002.tif]

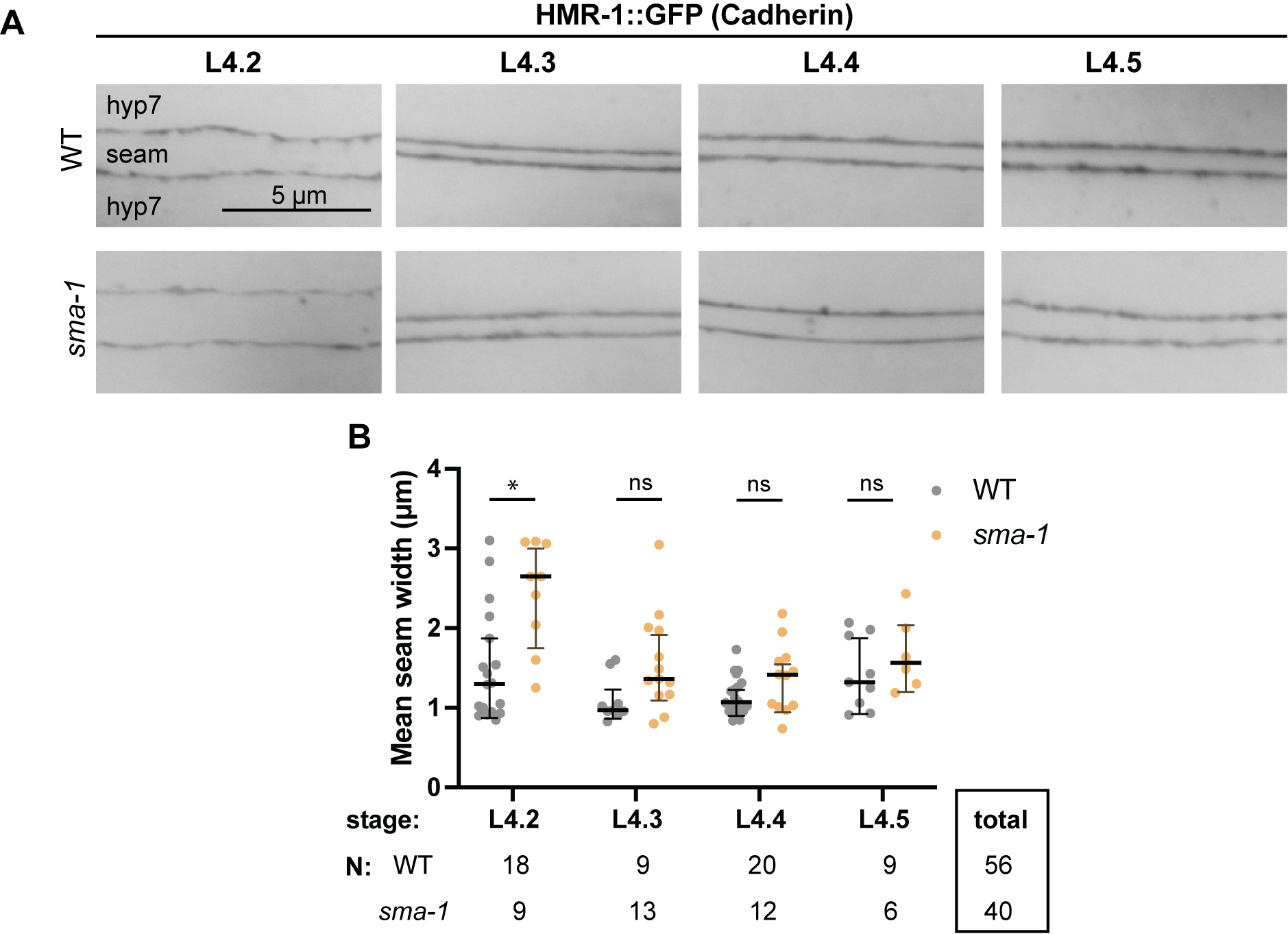

Supplement: S3 Fig — (A) Representative epifluorescence images of HMR-1::GFP (cadherin) marking apical junctions between the hyp7 and seam syncytia at mid-L4 substages. (B) Quantification of seam apical width calculated as the average of 6 measurements per worm taken 100px/9.6 um apart. Bars: mean and interquartile range. *p < 0.0125; Mann-Whitney U test with Bonferroni correction of 4 stages compared. (TIF) [file pgen.1012236.s003.tif]
