# Supplementary material for: Apical spectrin organizes cortical actin filament bundles to pattern C. elegans cuticle ridges
Source: PLoS Genet. 2026 Jul 16;22(7):e1012236. doi: 10.1371/journal.pgen.1012236 (PMC13395344; doi:10.1371/journal.pgen.1012236)
Supplement: S1 Table — (PDF) [file pgen.1012236.s009.pdf]

Table S1. Actin binding protein and CeHD fusion screening

Strain	Gene	Allele	Protein family	Seam signal at mid-L4	Reference
MT10865	*ced-10*	*nEx1039 [ced-10pro::GFP::CED-10]*	Rac	none detected	(Lundquist et al 2001)
BOX213	*erm-1*	*mib15 [ERM-1::GFP]*	Ezrin-Radixin-Moesin	AFB-like	(Remmelzwaal et al 2021)
CS4019	*ifb-1*	*juEx595 [IFB-1B::GFP + rol-6d]*	Intermediate filament	single medial band	(Woo et al 2004)
NK2324	*ina-1*	*qy23 [INA-1::GFP]*	integrin	seam-hyp border	(Jayadev et al 2019)
ML2501	*let-805*	*mc73 [LET-805::GFP + unc-119(+)]*	Myotactin	seam-hyp border	(Quintin et al 2016)
UP4337	*mup-4*	*mc120 [MUP-4::GFP + LoxP]*	Matrilin	single medial band (L4.6)	(Suman et al. 2019)
NK2479	*pat-2*	*qy49 [PAT-2::2xmNG]*	Integrin	none detected	(Jayadev et al 2019)
NK2436	*pat-3*	*qy36 [PAT-3::mNG]*	Integrin	none detected	(Jayadev et al 2019)
RZB213	*plst-1*	*msn190 [PLST-1::GFP]*	Plastin	seam-hyp border	(Ding et al 2017)
PHX4964	*sma-1*	*syb4954 [SMA-1::GFP]*	βH-spectrin	AFB-like	(Barker et al. 2023)
GOU2936	*spc-1*	*cas815 [SPC-1::GFP]*	α-spectrin	AFB-like	(Jia et al. 2019)
CZ25013	*unc-44*	*ju1413 [UNC-44::GFP::LoxP::3xFLAG)*	Ankyrin	seam-hyp border	(Chen et al 2017)
GOU3103	*unc-70*	*cas962 [GFP::UNC-70]*	β-spectrin	seam-hyp border	(Jia et al. 2019)
GOU2043	*vab-10*	*cas602 [VAB-10a::GFP]*	spectraplakin	single medial band	(Jia et al. 2019)
ML2799	*vab-10*	*mc123 [VAB-10b::GFP + LoxP]*	spectraplakin	single medial band	(Suman et al. 2019)
CZ3103	*vab-19*	*juIs167 [VAB-19::GFP + rol-6d]*	ankyrin repeat	seam-hyp border	(Ding et al 2003)
