# Supplementary material for: Apical spectrin organizes cortical actin filament bundles to pattern C. elegans cuticle ridges
Source: PLoS Genet. 2026 Jul 16;22(7):e1012236. doi: 10.1371/journal.pgen.1012236 (PMC13395344; doi:10.1371/journal.pgen.1012236)
Supplement: S2 Table — (PDF) [file pgen.1012236.s010.pdf]

Table S2: *vab-10* and *ifb-1b* reduced function mutants have normal alae

Strain	Gene	Allele	Alae Phenotype	N
CZ4380	*ifb-1b*	*ju71*	<10% partial disorg	20
N2	*vab-10*	*RNAi*	normal	22
N2	*vab-10a*	*RNAi*	normal	31
N2	*vab-10b*	*RNAi*	normal	15
CB698	*vab-10a*	*e698*	<10% partial disorg	24
ML2397	*vab-10*	*mc62*	normal	15
