# Supplementary material for: Apical spectrin organizes cortical actin filament bundles to pattern C. elegans cuticle ridges
Source: PLoS Genet. 2026 Jul 16;22(7):e1012236. doi: 10.1371/journal.pgen.1012236 (PMC13395344; doi:10.1371/journal.pgen.1012236)
Supplement: S3 Table — (PDF) [file pgen.1012236.s011.pdf]

Table S3. ***C. elegans*** **strains used in this study**

Strain	Genotype	Source and Reference
N2	*wild type (Bristol)*	Caenorhabditis Genetics Center (CGC)
ARF403	*aaaIs24 (elt-5p::untrnch::GFP::unc-54 3'UTR + ttx-3::GFP)*	Allison Frand Lab (Katz et al. 2022)
AZ30	*sma-1(ru18) V*	CGC (Praitis et al. 2005)
CB30	*sma-1(e30) V*	CGC (Praitis et al. 2005)
CB698	*vab-10(e698) I*	CGC (Bosher et al. 2003)
CZ4380	*ifb-1(ju71) II*	CGC (Woo et al 2004)
CZ25013	*unc-44(ju1413 [unc-44::gfp::LoxP::3xflag]) IV*	CGC (Chen et al 2017)
GCP808	*sma-1(prt133 [SMA-1(ΔPH)]) V*	Ana Carvalho Lab (Sobral et al., 2021)
GCP901	*sma-1(prt156 [SMA-1(ΔABD1)])V*	Ana Carvalho Lab (Sobral et al., 2021)
GCP986	*sma-1(prt163[SMA-1(ΔSH3)]) V*	Ana Carvalho Lab (Sobral et al., 2021)
GCP1102	*sma-1(prt192[SMA-1(ΔSR11)]) V*	Ana Carvalho Lab (Sobral et al., 2021)
GCP1138	*sma-1(prt185 [SMA-1(ΔABD2)]) V*	Ana Carvalho Lab (Sobral et al., 2021)
GOU2043	*vab-10a(cas602 [vab-10a::gfp]) I*	CGC (Jia et al. 2019)
GOU2936	*spc-1 (cas815 [spc-1::GFP)] X*	CGC (Jia et al. 2019)
GOU3103	*unc-70 (cas962 [GFP::unc-70]) V*	CGC (Jia et al. 2019)
GOU3237	*unc-70 (cas983) V*	CGC (Jia et al. 2019)
GOU3238	*spc-1 (cas971 (spc-1 [L268P]) X*	CGC (Jia et al. 2019)
ML2397	*vab-10(mc62 [DSH3_820-873]) I*	Michel Labouesse lab (Suman et al. 2019)<br>Partial loss of function *vab-10* allele.
ML2600	*vab-10(mc100[vab-10A::mCh + loxp]) I*	Michel Labouesse lab (Suman et al. 2019)
ML2764	*vab-10(mc109[vab.10(ΔSH3_820-873)::mCherry + LoxP] I ; mup-4(mc120 [mup-4::gfp + LoxP)] III*	Michel Labouesse lab (Suman et al. 2019)
ML2799	*VAB-10 (mc123 [vab-10B::GFP + LoxP] I*	Michel Labouesse lab (Suman et al. 2019)
PHX4954	*sma-1(syb4954 [sma-1::GFP]) V*	Ana Carvalho Lab (Barker et al. 2023)
UP2050	*unc-119(ed3); xnIs96 [pJN455::hmr-1::HMR-1::GFP::unc-54 3'UTR]; unc-119(+)*	Jeremy Nance Lab (Achilleos et al. 2010)<br>but renamed in the Sundaram Lab
UP4170	*lpr-3(cs266 [mCherry::LPR-3]) X ; aaaIs24 (elt-5pro::UTRNCH::GFP, ttx-3::GFP)*	Sundaram Lab (Katz et al. 2022)
UP4201	*vab-10(mc123 [vab-10B::GFP+LoxP] I; aaaIs21 [elt-5p::utrnch:dsred::unc-54 3'utr, ttx-3::dsred] 4x)*	This report
UP4207	*mc123 [vab-10A::mCherry+LoxP] I; aaaIs24 [elt-5p::utrnch::GFP::unc-54 3'UTR +ttx-3::GFP] 4x)*	This report
UP4240	*aaaIs24(egl-18p::untrnch::GFP::unc-54 3' UTR + ttx-3::GFP) II; sma-1(ru18) V*	This report
UP4241	*sma-1(ru18) V; spc-1(cas815[spc-1::GFP]) X*	This report
UP4242	*sma-1(ru18) V; lpr(cs250[ss::sfGFP::LPR-3]) X*	This report
UP4246	*vab-10(mc100[vab10a::mCh,LoxP]);juEx595(ifb-1B::GFP+pRF4[rol6d])*	This report
UP4247	*vab-10(mc100[vab-10a::mCh,LoxP]);spc-1(cas815[spc-1::GFP]) X*	This report
UP4256	*syb4954(sma-1::GFP); vab-10(mc100[vab10a::mCherry+loxP])*	This report
UP4252	*vab-10(cas602 [vab-10a::gfp]) I; sma-1(ru18) V*	Sundaram Lab (Barker et al. 2023)
UP4275	*aaaIs21 [egl-18pro::utrnch::dsred::unc-54 3' utr, ttx-3::dsred])]; sma-1(syb4954 [sma-1::gfp]) V*	This report
UP4333	*vab-10(cas602 [vab-10a::gfp]) I; sma-1(ru18) V*	This report
UP4336	*sma-1(ru18)V; unc-119(ed3);xnIs96[pJN455::hmr-1::HMR-1::GFP::unc-54 3'UTR]*	This report
UP4337	*mup-4(mc120 [mup-4::gfp + LoxP)] III*	This report<br>Derived from ML2764 (Suman et al. 2019)
UP4347	*vab-10(e698) I; sma-1(ru18) V*	This report
UP4366	*vab-10(e698) I/hT2[qIs48 [myo-2pro::GFP, let-?, bli-4(e937)] (I, III); spc-1 (cas971 (spc-1 [L268P]) X*	This report
UP4383	*rde-1(ne300) V; aaaIs4 (elt-5p::rde-1::SL-2::mCherry::unc-54 3'UTR + ttx-3::GFP) X*	This report
UP4405	*mfIs70 ([lin-31p::rde-1 + myo2p::GFP)] IV; rde-1(ne300) V*	This report
WM45	*rde-1(ne300) V*	CGC (Tabara et al. 1999)
